# Impact of polypharmacy on clinical outcomes in patients with advanced heart failure undergoing cardiac resynchronization therapy

**DOI:** 10.1002/joa3.13185

**Published:** 2024-11-22

**Authors:** Yuma Ono, Hidekazu Kondo, Taisuke Harada, Kunio Yufu, Hiroki Sato, Kazuki Mitarai, Keisuke Yonezu, Katsunori Tawara, Hidefumi Akioka, Naohiko Takahashi

**Affiliations:** ^1^ Department of Cardiology and Clinical Examination, Faculty of Medicine Oita University Yufu Oita Japan

**Keywords:** cardiac resynchronization therapy, cardiovascular outcome, heart failure with reduced ejection fraction, hyperpolypharmacy, survival analysis

## Abstract

**Background:**

The prevalence rates of heart failure (HF) and hyperpolypharmacy have increased with the aging population. While a negative impact of hyperpolypharmacy on HF clinical outcomes has already been reported, the effects of hyperpolypharmacy on patients with advanced HF with reduced ejection fraction (HFrEF) undergoing cardiac resynchronization therapy (CRT) remain unclear.

**Methods:**

We retrospectively evaluated data from 147 patients with advanced HFrEF who underwent CRT between March 2004 and June 2020. Patients were divided into nonpolypharmacy (<5 medications) and polypharmacy (≥5 medications) groups, as well as nonhyperpolypharmacy (<10 medications) and hyperpolypharmacy (≥10 medications) groups.

**Results:**

The mean age of the study population was 70.6 ± 9.7 years, and 90 patients (67.2%) were male. The median number of medications used was 10 (interquartile range: 7–13, range: 2–24); Kaplan–Meier survival analysis revealed that the hyperpolypharmacy group had a significantly worse long‐term survival rate in terms of major adverse cardiovascular events (MACE; *p* = 0.004) and all‐cause mortality (*p* = 0.005). Long‐term survival in terms of MACE and all‐cause mortality was not significantly different between the polypharmacy with cardiovascular medication and nonpolypharmacy with cardiovascular medication groups. By contrast, the polypharmacy with noncardiovascular medication group had a significantly worse long‐term survival rate in terms of MACE (*p* = 0.006) and all‐cause mortality (*p* = 0.003) than the nonpolypharmacy with noncardiovascular medication group.

**Conclusions:**

Hyperpolypharmacy was significantly associated with adverse cardiovascular outcomes in patients with advanced HFrEF who underwent CRT. Noncardiovascular polypharmacy may underlie the harmful effects of hyperpolypharmacy.

## Graphical abstract

Hyperpolypharmacy was significantly associated with clinical outcome in patients who underwent cardiac resynchronization therapy. Noncardiovascular (CV) polypharmacy, but not CV polypharmacy, may be related to the adverse effects of hyperpolypharmacy.

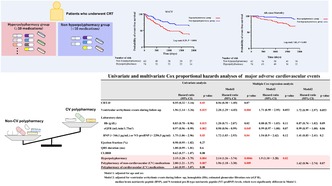

## 1 INTRODUCTION

The rates of chronic heart failure (HF) and polypharmacy have increased with the aging population and increase in comorbidities.^1^ A previous study based on a cross‐sectional analysis of community‐dispensed prescribing data reported that the proportions of prescriptions for five or more medications and 10 or more medications were larger in 2010 than in 1995 because of the aging of the population.^2^ Patients with HF with reduced ejection fraction (HFrEF) are more likely to have comorbidities and practice polypharmacy.^3^ Several studies have already reported on the association of hyperpolypharmacy with adverse clinical outcomes in patients with HF.^4^, ^5^, ^6^ However, the association between hyperpolypharmacy and clinical outcomes in patients with advanced HFrEF who require cardiac resynchronization therapy (CRT) remains unknown.

CRT is one of the treatment options for HFrEF patients with a wide QRS duration and poor improvement after guideline‐directed medical therapy (GDMT). Therefore, patients with HFrEF who undergo CRT are highly likely to have already undergone GDMT, which could be a possible cause of hyperpolypharmacy. We considered it relevant to explore whether hyperpolypharmacy exacerbates cardiovascular outcomes in this population. Therefore, in this study, we aimed to evaluate the association between hyperpolypharmacy and long‐term clinical outcomes in patients with HF who underwent CRT.

## 2 METHODS

### 2.1 Study design

**FIGURE 1:**
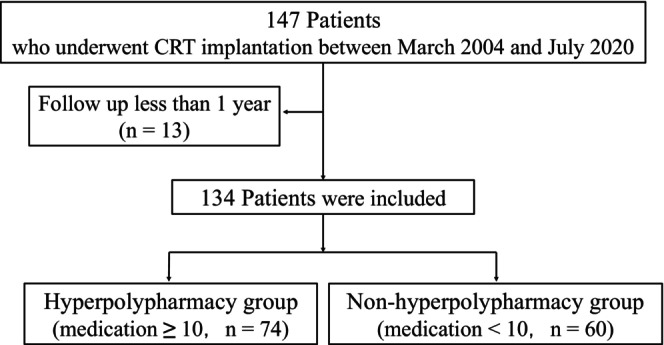
Flowchart of 147 consecutive patients undergoing CRT. CRT, cardiac resynchronization therapy.

This was a retrospective single‐center cohort study. The study protocol is illustrated in Figure 1. In total, 147 patients with chronic HF who underwent CRT between March 2004 and July 2020 were enrolled. We excluded patients with follow‐up periods of <1 year, and 134 patients were finally eligible for this study. First, the patients were divided into two groups according to the presence of hyperpolypharmacy (≥10 medications), and the effects of hyperpolypharmacy on the clinical outcomes were investigated. Second, to explore the effects of polypharmacy (≥5 medications) with cardiovascular medications (CV polypharmacy) or with noncardiovascular medications (non‐CV polypharmacy), the long‐term outcomes of patients with or without CV and non‐CV polypharmacy were also investigated (Figure S1).

We followed up the enrolled patients for more than 1 year and up to 5 years after the CRT implantation procedure. In this study, the indications for CRT adhered to the Japanese Circulation Society guidelines.^7^ CRT responders were defined as patients with over 15% reduction in the left ventricular end‐systolic volume (LVESV) at least 6 months after undergoing CRT.^8^ Patients with less than 15% reduction in the LVESV at least 6 months after undergoing CRT and patients who had been readmitted for HF, had a worsening NYHA class at the final follow‐up, had continuous moderate‐to‐severe deterioration of the clinical composite score,^9^ had stopped biventricular pacing due to worsening HF, or had died within 6 months after CRT were defined as nonresponders.^8^

### 2.2 Definition of polypharmacy and cardiovascular medications

Information on medications was collected from medical records at the time of discharge from hospitalization after CRT device implantation. Polypharmacy was defined as the prescription of ≥5 types of medications, and hyperpolypharmacy, as the prescription of ≥10 types of medications. These are the cutoffs most commonly used in previous studies.^1^, ^10^ If a patient was taking a fixed‐dose combination of two medicines, the medication was considered as two different medicines. The hyperpolypharmacy group was defined as patients taking a total of ≥10 medications at the time of discharge after CRT implantation. The polypharmacy with cardiovascular medication group was defined as patients taking a total of ≥5 medications prescribed for cardiovascular diseases at the time of discharge after CRT implantation. Similarly, the polypharmacy with noncardiovascular medication group was defined as patients taking a total of ≥5 medications prescribed for noncardiovascular diseases (Figure S1). For instance, patients taking six CV medications and six non‐CV medications were categorized in both CV polypharmacy and non‐CV polypharmacy groups. Angiotensin‐converting enzyme inhibitors (ACEIs), angiotensin‐II receptor blockers (ARBs), mineralocorticoid receptor antagonist (MRAs), beta‐blockers, diuretics (loop, thiazide, and tolvaptan), calcium channel blockers, digoxin, antiarrhythmics, pimobendane, HMG‐CoA reductase inhibitors (statins), and oral anticoagulants/antiplatelets were defined as CV medications, whereas all other drugs were defined as non‐CV medications.

Sacubitril/valsartan (an angiotensin receptor neprilysin inhibitor) was not included in the study, and the sodium glucose cotransporter 2 inhibitor canagliflozin was prescribed as an antidiabetic drug to only one patient.

### 2.3 Study outcomes

Information on patients' clinical outcomes was retrospectively obtained from medical records. The primary outcome of this study was major adverse cardiovascular events (MACE), including stroke, acute coronary syndrome, hospitalization for HF, and cardiovascular death. The secondary outcome was all‐cause mortality and ventricular arrhythmia events. Ventricular arrhythmia was defined as ventricular tachycardia or ventricular fibrillation requiring antitachycardia pacing or cardioversion.

### 2.4 Statistical analysis

Continuous data are expressed as mean ± standard deviation or as median with interquartile range. Analysis of variance was used for continuous variables, and the chi‐squared test was used for categorical variables. Student's *t*‐test was used to analyze differences between groups. The Wilcoxon test was used for nonparametric tests. Univariate Cox proportional hazards regression analyses were performed to distinguish factors predicting primary and secondary outcomes. Multiple Cox regression analysis was performed to evaluate independent risk factors for MACE using multiple models. In Model 1, we adjusted for age and gender. In Model 2, we adjusted for hemoglobin (Hb), estimated glomerular filtration rate (eGFR), median brain natriuretic peptide (BNP), and N‐terminal pro‐B‐type natriuretic peptide (NT‐proBNP), which exhibited significant differences in Model 1. In Model 3, we adjusted for gender, atrial fibrillation, ejection fraction (EF), QRS duration, and complete left bundle branch block (CLBBB) in addition to the factors in Model 2. Atrial fibrillation, CLBBB, and QRS duration have been reported to affect the response to CRT.^11^, ^12^, ^13^ Cumulative incidences were estimated using the Kaplan–Meier method and compared using the log‐rank test. The results were presented as hazard ratios (HRs) with 95% confidence intervals (CIs). Statistical significance was set at *p* < 0.05. All analyses were performed using JMP software (version 11.2.0; SAS Institute, Cary, NC, USA).

## 3 RESULTS

### 3.1 Patient characteristics

**FIGURE 2:**
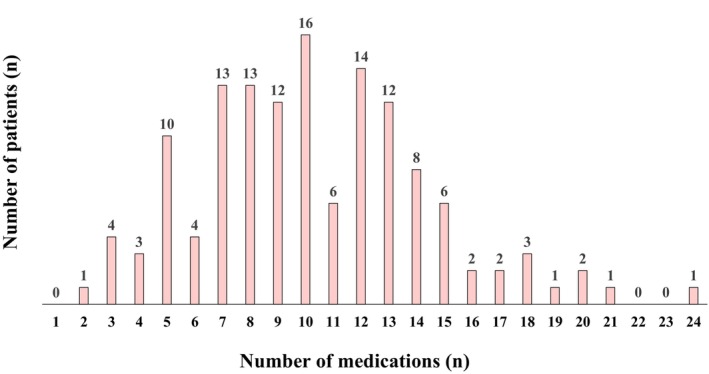
Distribution of the total number of medications and of patients who were taking each number of medications.

The mean age of the total population was 70.6 ± 9.7 years, and 90 patients (67.2%) were male. The distribution of the total number of medications ranged from 2 to 24, and the number of patients prescribed each number of medication is shown in Figure 2. The median number of medications used was 10 (interquartile range, 7–13). Table 1 presents the baseline characteristics of the study participants. Male gender, NYHA stage IV, and the presence of diabetes were significantly more common in the hyperpolypharmacy group, whereas the presence of CLBBB and CRT responders was significantly less common. In terms of blood laboratory findings, significantly higher CRP and lower total protein and albumin levels were observed in the hyperpolypharmacy group. In the hyperpolypharmacy group, dilated cardiomyopathy was significantly less common, and ischemic cardiomyopathy was significantly more common as an etiology of HF.

**TABLE 1:** Baseline characteristics of the entire study population and the hyperpolypharmacy and nonhyperpolypharmacy groups.

	Total population	Nonhyperpolypharmacy	Hyperpolypharmacy	*p*‐alue
	(*n =* 134)	(*n =* 60)	(*n =* 74)	
Age (years)	72 [65–78]	70.5 [64–77]	73 [66–78]	.37
Gender, male	90 (67.1%)	34 (56.7%)	56 (75.7%)	.02
Body height (cm)	158.8 ± 9.9	158.0 ± 10.2	159.4 ± 9.7	.61
Body weight (kg)	57.9 ± 12.1	57.1 ± 11.7	58.5 ± 12.4	.6
Body mass index (kg/m^2^)	23.0 [19.4–25.0]	22.4 [20.0–25.0]	23.2 [19.9–24.8]	.88
CRT‐D	108 (80.6%)	47 (78.3%)	61 (82.4%)	.55
CRT‐P	26 (19.4%)	13 (21.7%)	13 (19.4%)	.55
NYHA				
II	54 (40.3%)	26 (43.3%)	28 (37.8%)	.52
III	62 (46.3%)	31 (51.7%)	32 (43.2%)	.33
IV	18 (13.4%)	3 (5.0%)	15 (20.3%)	.01
III or IV	80 (59.7%)	34 (56.7%)	46 (62.2%)	.52
Past medical history				
Hypertension	47 (35.1%)	22 (36.7%)	25 (33.8%)	.73
Diabetes	47 (35.1%)	12 (20.0%)	35 (47.3%)	.001
Dyslipidemia	63 (47.0%)	24 (40.0%)	39 (52.7%)	.14
Cerebrovascular disease	10 (7.5%)	5 (8.3%)	5 (6.8%)	.73
Atrial fibrillation	49 (36.6%)	20 (33%)	29 (39.1%)	.48
VT or VF	37 (27.6%)	12 (20%)	25 (33.8%)	.08
Etiology of HF				
DCM	57 (42.5%)	32 (56.1%)	25 (43.9%)	.02
ICM	31 (23.1%)	6 (10.0%)	25 (33.8%)	.001
HCM	4 (3.0%)	2 (3.3%)	2 (2.7%)	.83
Sarcoidosis	21 (15.7%)	10 (16.7%)	11 (14.9%)	.77
Valvular disease	8 (6.0%)	4 (6.7%)	4 (5.4%)	.76
Others	13 (9.7%)	6 (10%)	7 (9.5%)	.92
Laboratory data				
Hb (g/dL)	12.5 ± 1.7	12.7 ± 1.7	12.3 ± 1.7	.16
CRP (mg/dL)	0.16 [0.06–0.44]	0.09 [0.05–0.22]	0.26 [0.08–0.51]	.004
TP (g/dL)	6.8 ± 0.68	7.0 ± 0.09	6.7 ± 0.71	.04
Alb (g/dL)	3.8 ± 0.46	3.9 ± 0.41	3.7 ± 0.49	.02
T‐Bil (md/dL)	0.78 [0.59–1.03]	0.78 [0.6–1.05]	0.78 [0.57–1.03]	.71
AST (U/L)	23.3 [19.8–31.6]	23.2 [18.3–32.5]	23.4 [20.0–29.3]	.89
ALT (U/L)	16.9 [12–24.2]	15.8 [11.9–23.5]	17.7 [12.2–24.3]	.51
eGFR (mL/min/1.73m^2^)	45.9 [35.1–58.0]	49.0 [38.7–60.4]	42.9 [34.9–54.4]	.06
BNP (pg/mL)	352.6 [173.8–676.3]	344.2 [151.7–594.6]	369.5 [207.8–769]	.5
NT‐proBNP (pg/mL)	2296.5 [887.5–4375.5]	2809.5 [655.7–5927]	2296.5 [1316.5–4374.5]	.95
Echocardiographic data				
Ejection fraction (%)	31.4 ± 8.2	31.1 ± 7.6	31.7 ± 8.7	.62
LVEDV (mL)	151 [118–208]	150 [118–204]	153 [123–213]	.56
LVESV (mL)	105 [76–147]	105 [77–143]	107 [76–152]	.9
QRS duration (ms)	166.2 ± 27.0	164.1 ± 25.7	167.9 ± 28.0	.38
CRT responder	75 (56.0%)	41 (68.3%)	34 (46.6%)	.01
CLBBB	95 (70.9%)	48 (80.0%)	47 (63.5%)	.04

*Note*: Data are presented as the mean ± standard deviation, median [interquartile range], or number (percentage).

Abbreviations: Alb, albumin; ALT, alanine aminotransferase; AST, aspartate aminotransferase; BNP, brain natriuretic peptide; CLBBB, complete left bundle branch block; CRP, C‐reactive protein; CRT‐D, cardiac resynchronization therapy with defibrillator; CRT‐P, cardiac resynchronization therapy without defibrillator; DCM, dilated cardiomyopathy; eGFR, estimated glomerular filtration rate; Hb, hemoglobin; HCM, hypertrophic cardiomyopathy; HF, heart failure; ICM, ischemic cardiomyopathy; LVEDV, left ventricular end‐diastolic volume; LVESV, left ventricular end‐systolic volume; NT‐proBNP, N‐terminal pro‐brain natriuretic peptide; NYHA, New York Heart Association Functional Classification; T‐bil, total bilirubin; TP, total protein; VF, ventricular fibrillation; VT, ventricular tachycardia.

Regarding medications (Table 2), the numbers of overall, cardiovascular, and noncardiovascular medications were significantly greater in the hyperpolypharmacy group. Among cardiovascular medicines, the numbers of prescriptions for loop diuretics, tolvaptan, pimobendane, antiarrhythmics, antithrombotic drugs, and HMG‐CoA reductase inhibitors were significantly higher in the hyperpolypharmacy group. In terms of noncardiovascular medicines, the numbers of prescriptions for antidiabetics, gastric mucosal protectant drugs, laxatives, and minor tranquilizers or sleep medications were significantly higher in the hyperpolypharmacy group.

**TABLE 2:** Medication at discharge in the entire study population and the hyperpolypharmacy and nonhyperpolypharmacy groups.

	Total population	Nonhyperpolypharmacy	Hyperpolypharmacy	*p*‐value
	(*n =* 134)	(*n =* 60)	(*n =* 74)	
Total number of medications	10 [7–13]	7 [5–8]	13 [11–14.3]	.005
Total number of cardiovascular medications	5 [4–7]	4 [3–5]	7 [5–8]	<.001
Total number of noncardiovascular medications	4 [3–6]	3 [1–4]	6 [5–8]	<.001
Cardiovascular medications				
ACEIs or ARBs	109 (81.3%)	47 (78.3%)	62 (83.8%)	.42
ACEIs	60 (44.8%)	27 (45%)	33 (44.6%)	.96
ARBs	51 (38.0%)	21 (35%)	30 (40.5%)	.51
Beta‐blockers	125 (93.3%)	56 (93.3%)	69 (93.2%)	.98
MRAs	90 (67.2%)	35 (58.3%)	55 (74.3%)	.05
Calcium channel blockers	15 (11.2%)	7 (11.7%)	8 (10.8%)	.88
Loop diuretics	102 (76.1%)	38 (63.3%)	64 (86.5%)	.01
Thiazide diuretics	2 (1.5%)	0	2 (2.7%)	.21
Tolvaptan	28 (20.9%)	4 (6.7%)	24 (32.4%)	<.001
Digoxin	9 (6.7%)	3 (5%)	6 (8.1%)	.47
Pimobendane	22 (16.4%)	5 (8.3%)	17 (23.0%)	.02
Amiodarone	69 (51.5%)	24 (40.0%)	45 (60.8%)	.02
Antidysrhythmics other than amiodarone	10 (7.5%)	1 (1.7%)	9 (12.2%)	.02
Antiplatelet medications	35 (26.1%)	9 (15%)	26 (35.1%)	.01
Anticoagulant medications	78 (58.2%)	27 (45%)	51 (68.9%)	.01
HMG‐CoA reductase inhibitors (statins)	60 (44.8%)	21 (35.0%)	39 (52.7%)	.04
Noncardiovascular medications				
Diabetes medications	28 (20.9%)	4 (6.7%)	24 (32.4%)	.002
Iron preparations	20 (14.9%)	7 (11.7%)	13 (17.6%)	.34
Gastric mucosal protectants	108 (80.6%)	41 (68.3%)	67 (90.5%)	.001
Laxatives	40 (29.9%)	5 (8.3%)	35 (47.3%)	<.001
Sleeping pills	49 (36.6%)	12 (20%)	37 (50.0%)	<.001

Abbreviations: ACEI, angiotensin‐converting enzyme inhibitor; ARB, angiotensin II receptor blocker; HMG‐CoA, 3‐hydroxy‐3‐methylglutaryl coenzyme A; MRA, mineralocorticoid receptor antagonist.

### 3.2 Clinical outcomes

**FIGURE 3:**
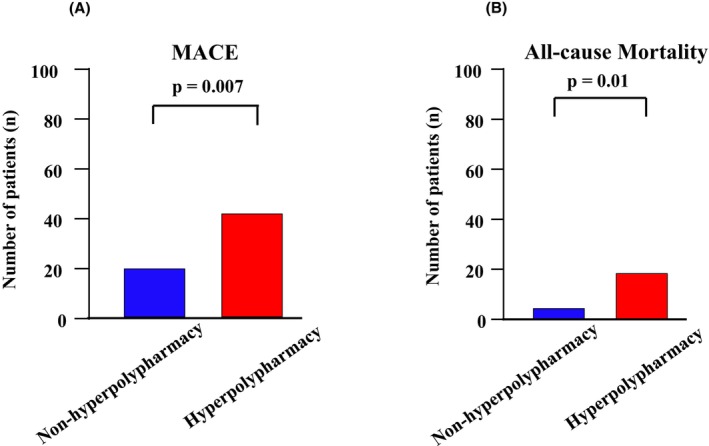
Comparison of MACE (A) and all‐cause mortality (B) between the hyperpolypharmacy and nonhyperpolypharmacy groups. MACE, major adverse cardiovascular event.

**FIGURE 4:**
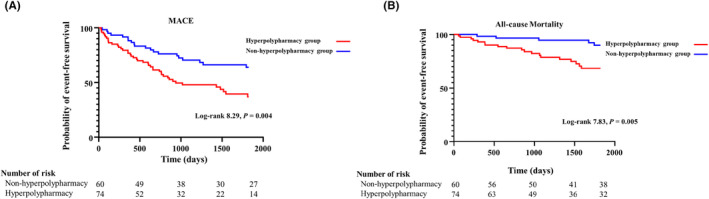
Kaplan–Meier survival analysis and log‐rank test for MACE (A) and all‐cause mortality (B) comparing the hyperpolypharmacy and nonhyperpolypharmacy groups. MACE, major adverse cardiovascular event.

The median follow‐up period was 1825 days (interquartile range, 873–1825 days). During the follow‐up period, MACE occurred in 62 patients (46%), ventricular arrhythmias occurred in 31 patients (23%), and 15 patients (11%) required cardioversion. All defibrillations were properly activated. The prevalence of MACE (*p* = 0.007, Figure 3A) and all‐cause mortality (*p* = 0.01, Figure 3B) were significantly higher in the hyperpolypharmacy group than in the nonhyperpolypharmacy group. The Kaplan–Meier survival analysis and the log‐rank test revealed that the hyperpolypharmacy group had a significantly worse long‐term survival rate in terms of MACE (log‐rank 8.29, *p* = 0.004; Figure 4A) and all‐cause mortality (log‐rank 7.83, *p* = 0.005; Figure 4B). In terms of ventricular arrhythmias, the two groups did not differ significantly (log‐rank 1.06, *p* = 0.30; Figure S2).

**FIGURE 5:**
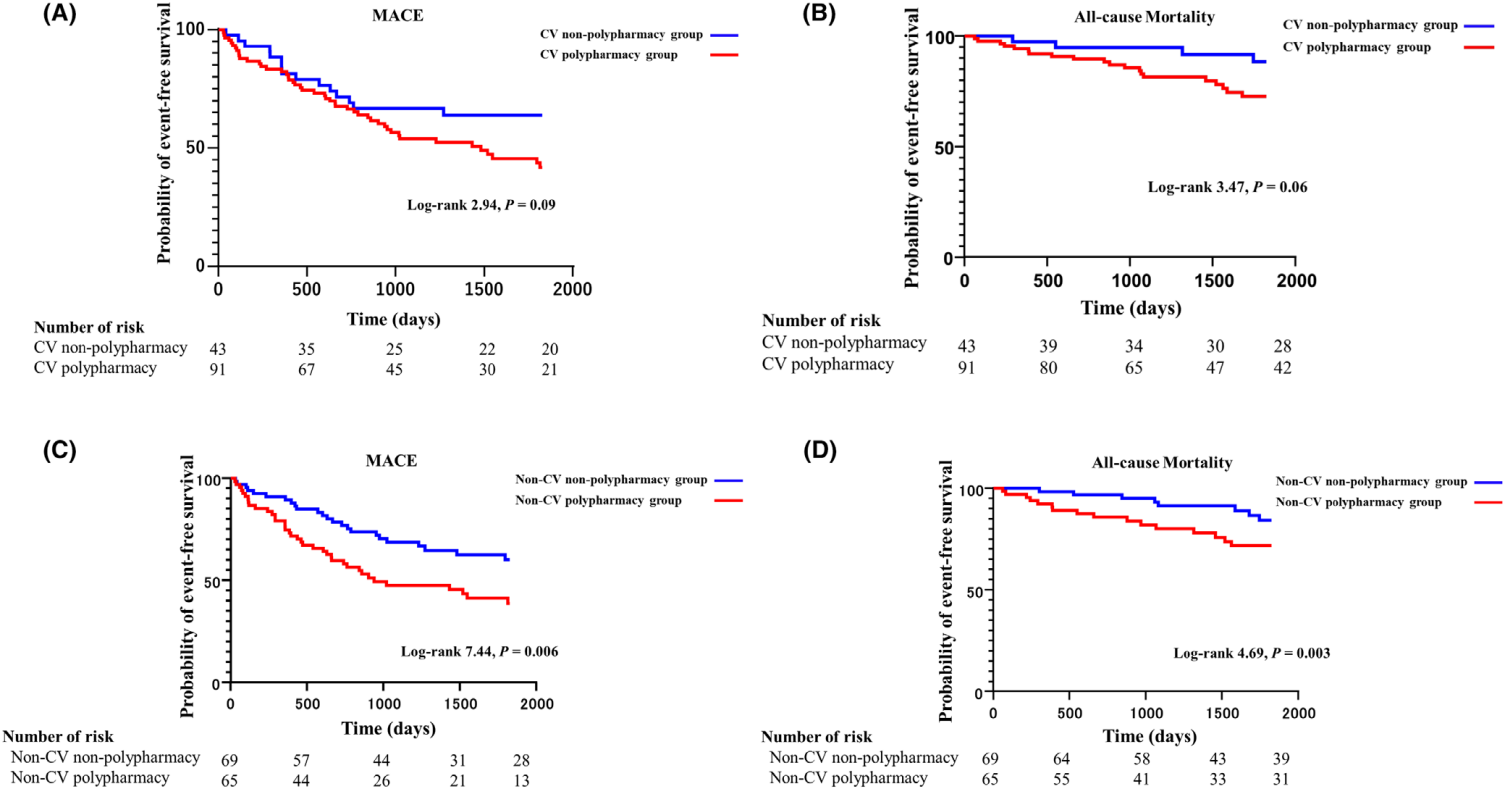
Kaplan–Meier survival analysis and log‐rank test comparing MACE (A) and all‐cause mortality (B) between the CV polypharmacy and CV nonpolypharmacy groups and comparing MACE (C) and all‐cause mortality (D) between the non‐CV polypharmacy and non‐CV nonpolypharmacy groups. CV, cardiovascular; MACE, major adverse cardiovascular event.

**FIGURE 6:**
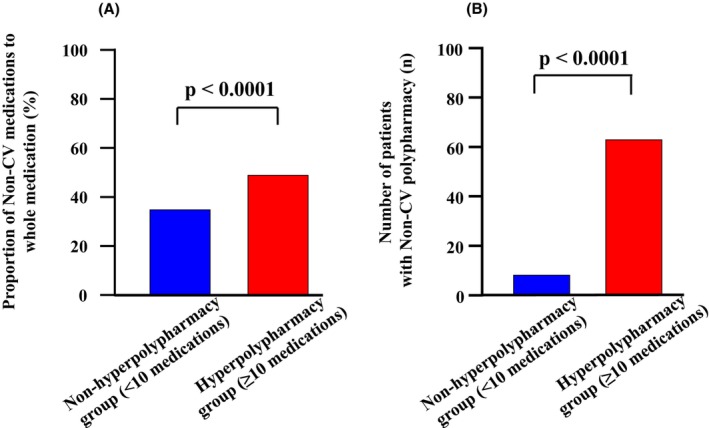
Comparison of the proportions of non‐CV medications among total medications (A) and numbers of patients practicing non‐CV polypharmacy (B) between the hyperpolypharmacy and nonhyperpolypharmacy groups. CV, cardiovascular.

Kaplan–Meier survival analysis and the log‐rank test revealed that there were no significant differences in long‐term survival rate in terms of MACE (log‐rank 2.94, *p* = 0.09; Figure 5A) and all‐cause mortality (log‐rank 3.47, *p* = 0.06; Figure 5B) between the CV polypharmacy and CV nonpolypharmacy groups, whereas the non‐CV polypharmacy group had a significantly worse long‐term survival rate in terms of both MACE (log‐rank 7.44, *p* = 0.006; Figure 5C) and all‐cause mortality (log‐rank 4.69, *p* = 0.003; Figure 5D) than the non‐CV nonpolypharmacy group. Supporting the validity of this finding, the proportion of non‐CV medications among overall medications in the hyperpolypharmacy group was significantly larger than that in the nonhyperpolypharmacy group (Figure 6A). Additionally, the number of patients practicing non‐CV polypharmacy was significantly higher in the hyperpolypharmacy group (Figure 6B).

Univariate and multivariate Cox proportional hazards analyses revealed that hyperpolypharmacy, particularly polypharmacy with noncardiovascular medications, was significantly associated with higher incidences of MACE, whereas polypharmacy with cardiovascular medications was not associated with MACE (Table 3).

**TABLE 3:** Univariate and multivariate Cox proportional hazards analyses of major adverse cardiovascular events.

	Univariate analysis		Multiple cox regression analysis					
			Model 1		Model 2		Model 2	
	HR (95% CI)	*p*‐value	HR (95% CI)	*p*‐value	HR (95% CI)	*p*‐value	HR (95% CI)	*p*‐value
Age (years)	1.02 (0.99–1.04)	.23						
Gender (male)	1.10 (0.66–1.90)	.72						
Body mass index (kg/m^2^)	0.96 (0.90–1.02)	.23						
NYHA III	1.37 (0.83–2.25)	.22						
NYHA IV	1.77 (0.94–3.33)	.08						
CRT‐D	0.55 (0.32–3.16)	.03	0.56 (0.30–1.05)	.07				
Ventricular arrhythmic events during follow‐up	1.96 (1.14–3.36)	.015	2.28 (1.29–4.03)	.004	1.71 (0.99–2.95)	0.053	1.72 (0.99–2.97)	.053
**Past medical history**								
Hypertension	0.78 (0.44–1.31)	.35						
Diabetes	1.20 (0.71–1.98)	.5						
Dyslipidemia	0.75 (0.45–1.24)	.26						
Cerebrovascular disease	1.90 (0.73–4.09)	.17						
Atrial fibrillation	1.30 (0.78–2.15)	.31						
VT or VF	1.16 (0.66–1.96)	.59						
**Etiology of HF**								
ICM	0.96 (0.51–1.69)	.88						
**Laboratory data**								
Hb (g/dL)	0.83 (0.70–0.96)	.015	1.20 (0.71–2.07)	.02	0.88 (0.75–1.03)	0.11	0.87 (0.74–1.02)	.09
CRP (mg/dL)	1.06 (0.81–1.28)	.62						
Alb (g/dL)	0.66 (0.39–1.14)	.14						
eGFR (mL/min/1.73m^2^)	0.97 (0.96–0.99)	.002	0.98 (0.96–0.99)	.045	0.99 (0.97–1.00)	0.07	0.99 (0.97–1.00)	.06
BNP (>346.1 pg/mL) or NT‐proBNP (>2296.5 pg/mL)	1.75 (1.06–2.96)	.03	1.72 (1.03–2.93)	.04	1.54 (0.9–2.62)	0.12	1.41 (0.83–2.41)	.2
Ejection fraction (%)	0.98 (0.95–1.02)	.27						
QRS duration (ms)	1.00 (0.99–1.01)	.6						
CLBBB	0.62 (0.37–1.07)	.08						
Hyperpolypharmacy	2.15 (1.28–3.75)	.004	2.14 (1.26–3.74)	.0046	1.9 (1.10–3.28)	0.02		
Polypharmacy with noncardiovascular medications	2.00 (1.21–3.37)	.007	1.96 (1.18–3.30)	.009			1.62 (0.96–2.74)	.07
Polypharmacy with cardiovascular medications	1.66 (0.95–3.07)	.08						

*Note*: Model 1: adjusted for age and gender. Model 2: adjusted for ventricular arrhythmic events during follow‐up, Hb, eGFR, and median BNP and NT‐proBNP levels, which were significantly different in Model 1.

Abbreviations: Alb, albumin; BNP, brain natriuretic peptide; CI, confidence interval; CLBBB, complete left bundle branch block; CRP, C‐reactive protein; CRT‐D, cardiac resynchronization therapy with defibrillator; eGFR, estimated glomerular filtration rate; Hb, hemoglobin; HF, heart failure; HR, hazard ratio; ICM, ischemic cardiomyopathy; NT‐proBNP, N‐terminal pro‐brain natriuretic peptide; NYHA, New York Heart Association Functional Classification; VF, ventricular fibrillation; VT, ventricular tachycardia.

Furthermore, the effects of the presence of antiplatelet and anticoagulant medications on all‐cause mortality and MACE were evaluated. In our study, 40 patients did not take any antithrombotic agents (antiplatelet agents and anticoagulants), whereas 19 patients were treated with dual antithrombotic agents. There were no significant differences in MACE or all‐cause mortality between the two groups (Figure S3). Furthermore, Cox proportional hazards were used to evaluate the effect of each medication (antiplatelet, anticoagulant, and dual antithrombotic medications), but all of these medications did not have significant effects on MACE or all‐cause mortality (Table S1).

## 4 DISCUSSION

In this study, we uncovered the following novel findings: (1) hyperpolypharmacy was a significant prognostic factor for MACE and all‐cause mortality, even in patients with advanced HF who underwent CRT; and (2) a separate analysis of non‐CV and CV polypharmacy showed that only non‐CV polypharmacy had an impact on the development of cardiovascular events. Our study included only patients with advanced HFrEF who required CRT and explored the association of polypharmacy with not only all‐cause mortality but also MACE in the long term (5 years), which is one of the novelties of our study.

Previous studies have reported that hyperpolypharmacy was associated with the cumulative 1‐year incidence of a composite of death or rehospitalization, which increased incrementally with an increasing number of medications,^5^ and also with an elevated risk of death in elderly patients after acute decompensated HF,^6^ which is consistent with the findings of our study. The mechanism underlying the association between the number of medications used and cardiovascular events in patients with HF remains to be elucidated. Deteriorated systemic conditions, medication adherence, and drug interactions may contribute to a worse prognosis. A previous study reported that patients practicing hyperpolypharmacy are more likely to have a variety of comorbidities, which often reflects an advanced phase of systemic illness.^6^ It is likely that the practice of hyperpolypharmacy reflects an advanced phase of systemic illness and leads to adverse clinical outcomes, which suggests that hyperpolypharmacy implies more deteriorated systemic conditions. The use of diuretics, pimobendane, and antiarrhythmics was higher in the hyperpolypharmacy group in our study, which is consistent with the aforementioned view. Previous studies have reported poor medication adherence and drug interactions in patients with HF taking more medications than in those taking fewer medications.^14^, ^15^ It has been reported that patients with HF who take more medications have a higher risk of adverse medication events than those who take fewer medications.^16^ These studies suggest that polypharmacy itself may exacerbate systemic conditions. These mechanisms may account for the poor prognosis observed in the present study.

With regard to the adverse effects of non‐CV polypharmacy on the hard endpoints, a previous study reported that benzodiazepines are associated with a higher risk of hospitalization for HF.^17^ Because insomnia is associated with adverse prognosis in patients with HF,^18^ it should be treated without benzodiazepines. In our study, 49 patients (36.6%) with comorbid insomnia required sleeping pills. Of these, 28 (57.1%) patients were taking benzodiazepines. Comorbid insomnia requiring sleeping pills was significantly associated with a higher MACE incidence in our study (HR: 2.39, 95% CI: 1.44–3.97, *p* = 0.001). Based on these findings, treatment of insomnia with benzodiazepines might have contributed to adverse outcomes.

In terms of CV medications, it is well‐established that GDMT with cardiovascular medications is important for patients with HFrEF. Previous studies have revealed that patients with HFrEF who could not be initiated or continued on beta‐blockers or ACE inhibitors at discharge had a higher mortality rate at 1 year than those who could be^19^, ^20^ and that beta‐blockers also improved the long‐term prognosis in patients with HFrEF and a pacemaker rhythm.^21^ Based on our study, no significant differences in MACE and all‐cause mortality were found between the CV polypharmacy and CV nonpolypharmacy groups. We speculate that this was because the use of diuretics, pimobendane, and antiarrhythmics was higher in the CV polypharmacy group than in the CV nonpolypharmacy group (data not shown). These medications have not been proven to improve the hard endpoints in patients with HF.

Further studies are required to demonstrate whether a reduction in non‐CV polypharmacy can lead to an improvement in the hard endpoints in patients with advanced HF.

### 4.1 Limitations

This study has several limitations. First, the medication dosage was not considered. Second, we did not follow‐up on the adherence to medications and changes in medication after discharge. Third, possible prognostic factors were entered into the adjusted models in our study; however, it should be noted that there were other possible relevant covariates.

## 5 CONCLUSION

Hyperpolypharmacy was significantly associated with MACE and all‐cause mortality in patients with advanced HFrEF who underwent CRT. Non‐CV polypharmacy, but not CV polypharmacy, may be related to the adverse effects of hyperpolypharmacy.

## FUNDING INFORMATION

The authors received no funding for this study.

## CONFLICT OF INTEREST STATEMENT

The authors declare that there are no conflicts of interest.

## ETHICS STATEMENT

The study was conducted in accordance with the ethics review board of Oita University. All procedures were performed in accordance with the principles of the Declaration of Helsinki of 1964.

## INFORMED CONSENT

Written informed consent was obtained from all study participants.

## Supporting information


Data S1.

Figure S1.

Table S1.

## Data Availability

Data supporting the findings of this study are available from the corresponding author upon request.
