# Supplementary material for: Impact of polypharmacy on clinical outcomes in patients with advanced heart failure undergoing cardiac resynchronization therapy
Source: J Arrhythm. 2024 Nov 22;41(1):e13185. doi: 10.1002/joa3.13185 (PMC11730717; doi:10.1002/joa3.13185)
Supplement: Supplementary file 1 — Data S1. [file JOA3-41-e13185-s003.docx]

**Supplementary Figure legends**

**Figure 1.** Flowchart for classifying patients with and without CV polypharmacy and patients with and without non-CV polypharmacy.

CV, cardiovascular; CRT, cardiac resynchronization therapy

**Supplementary Figure 2.** Kaplan-Meier survival analysis and log-rank test for occurrence of ventricular arrhythmic events between the hyperpolypharmacy and non-hyperpolypharmacy groups.

**Supplementary Figure 3.** Kaplan-Meier survival analysis and log-rank test for MACE (**A**) and all-cause mortality (**B**) between the patients who were taking dual antithrombotic agent (antiplatelet agent and anticoagulant) and those who were not taking any antithrombotic agent.

MACE, major adverse cardiovascular event
