# Supplementary material for: Impact of polypharmacy on clinical outcomes in patients with advanced heart failure undergoing cardiac resynchronization therapy
Source: J Arrhythm. 2024 Nov 22;41(1):e13185. doi: 10.1002/joa3.13185 (PMC11730717; doi:10.1002/joa3.13185)
Supplement: Supplementary file 2 — Figure S1. [file JOA3-41-e13185-s001.pptx]

## Slide 1
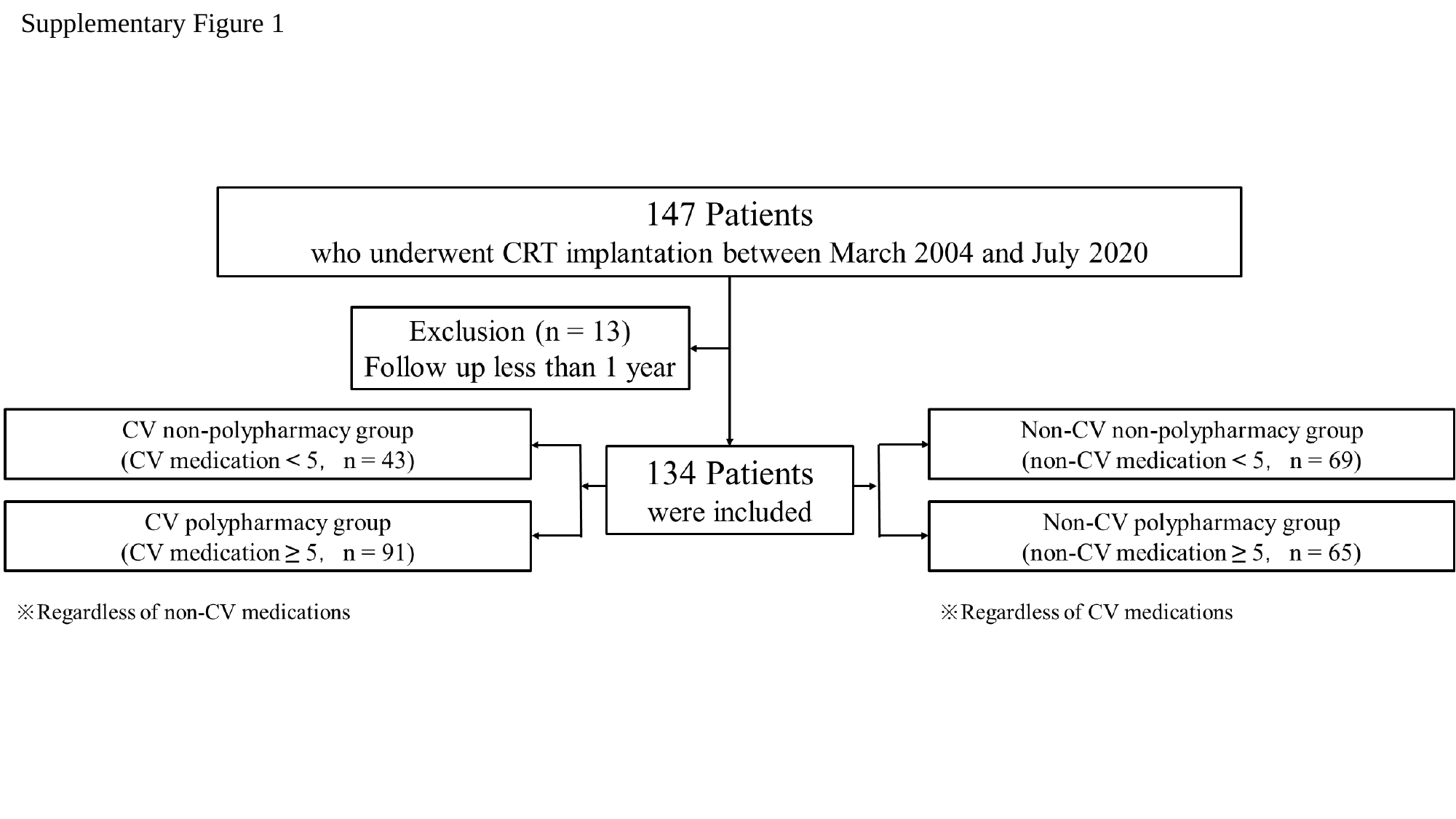

Supplementary Figure 1

## Slide 2
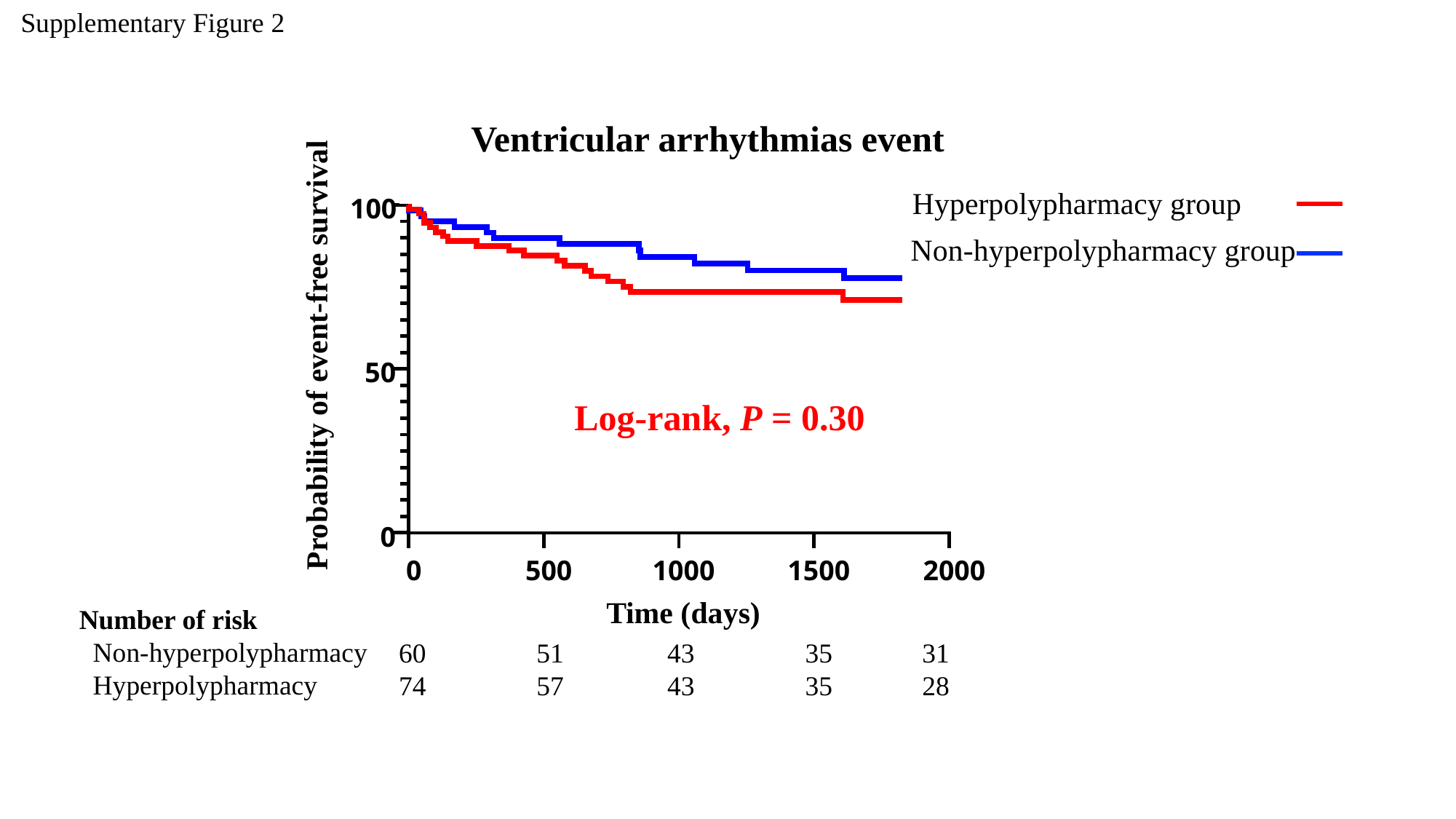

Supplementary Figure 2
Ventricular arrhythmias event
Hyperpolypharmacy group
100
Non-hyperpolypharmacy group
50
0
0
500
1000
1500
2000
Probability of event-free survival
Log-rank, P = 0.30
Time (days)
Number of risk
 Non-hyperpolypharmacy
 Hyperpolypharmacy
60 51 43 35 31
74 57 43 35 28

## Slide 3
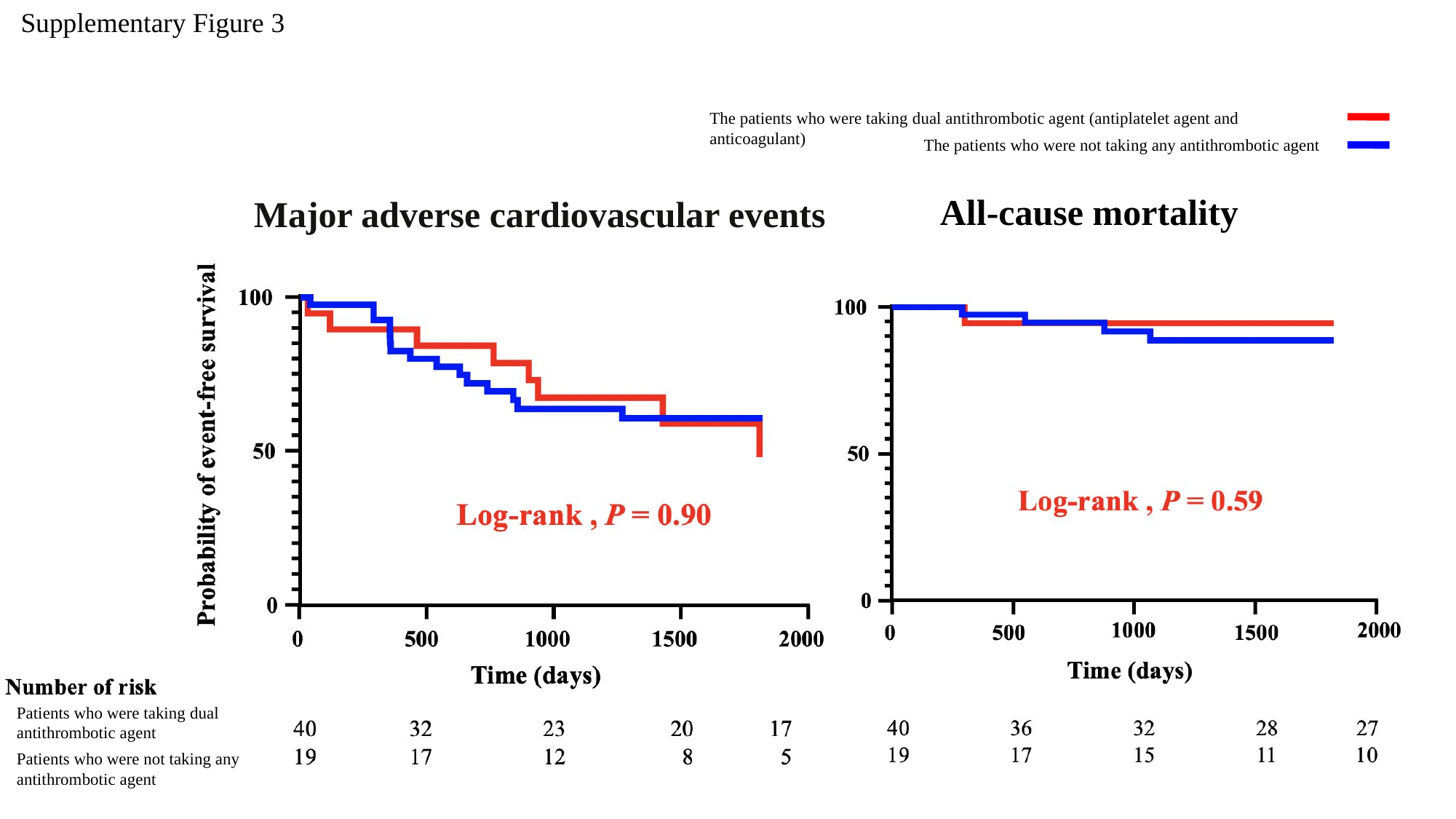

Supplementary Figure 3
The patients who were taking dual antithrombotic agent (antiplatelet agent and anticoagulant)
The patients who were not taking any antithrombotic agent
 All-cause mortality
 Major adverse cardiovascular events
Patients who were taking dual antithrombotic agent
Patients who were not taking any antithrombotic agent
