# Supplementary material for: Impact of polypharmacy on clinical outcomes in patients with advanced heart failure undergoing cardiac resynchronization therapy
Source: J Arrhythm. 2024 Nov 22;41(1):e13185. doi: 10.1002/joa3.13185 (PMC11730717; doi:10.1002/joa3.13185)
Supplement: Supplementary file 3 — Table S1. [file JOA3-41-e13185-s002.docx]

Supplementary Table 1

Univariate Cox proportional hazards analysis of major adverse cardiovascular events and all-cause mortality for the evaluation of the impact of antiplatelet medication, anticoagulant medication and dual antithrombotic medication

|  | **Univariate analysis of**  **major adverse cardiovascular events** | | **Univariate analysis of**  **all-cause mortality** | |
| --- | --- | --- | --- | --- |
|  | **Hazard ratio (95% CI)** | **p value** | **Hazard ratio (95% CI)** | **p value** |
| **Antiplatelet medications** | **0.86 (0.48 − 1.54)** | **0.61** | **0.79 (0.29 − 2.12)** | **0.64** |
| **Anticoagulant medications** | **1.39 (0.83 − 2.35)** | **0.21** | **1.55 (0.66 − 3.63)** | **0.31** |
| **Dual antithrombotic medications** | **0.79 (0.38 − 1.66)** | **0.53** | **0.27 (0.04 − 1.98)** | **0.20** |
